# Mothers with justice‐involved sons: Socioeconomic impacts of COVID‐19 by neighborhood disorder in the United States

**DOI:** 10.1111/josi.12527

**Published:** 2022-07-19

**Authors:** Alyssa LaBerge, Amanda Isabel Osuna, Caitlin Cavanagh, Elizabeth Cauffman

**Affiliations:** ^1^ School of Criminal Justice Michigan State University East Lansing Michigan USA; ^2^ Department of Psychological Science University of California Irvine California USA

## Abstract

Women, particularly mothers, have faced disparate socioeconomic consequences throughout the COVID‐19 pandemic. Research has yet to examine whether the consequences of the pandemic vary based on the level of neighborhood disorder, which is associated with various health conditions, including COVID‐19 complications. The present study utilizes data from a diverse sample of 221 women with justice‐involved sons interviewed during the early months of the COVID‐19 pandemic. Negative binominal and logistic regression analyses were conducted to examine whether perceived neighborhood social disorder is related to socioeconomic impacts of the COVID‐19 pandemic, and whether the relation varies for mothers with and without children in their home during the pandemic. The results suggest that greater perceived neighborhood social disorder was associated with increased in COVID‐19‐related socioeconomic consequences. Neighborhood social disorder affected socioeconomic impacts above and beyond the effects of having a child living in the home. Additionally, Latinas experienced greater socioeconomic impacts than women of other races and ethnicities. The results indicate a key relationship between the neighborhood conditions a woman lives in and the extent of the socioeconomic consequences they faced during the early months of the COVID‐19 pandemic. Future directions and direct implications of the study findings are discussed.

## JUSTICE‐INVOLVED MOTHERS: DIRECT SOCIOECONOMIC IMPACTS OF COVID‐19 BY NEIGHBORHOOD DISORDER IN THE UNITED STATES

The impacts of the COVID‐19 pandemic have not been equally distributed throughout the United States. For example, racial and ethnic minorities and lower socioeconomic classes in the United States have been impacted the most by health disparities and food and financial insecurities caused by the pandemic (Alkon et al., 2020; Jetha et al., 2021; Parker et al., 2020; Versey, 2022). Areas of increased poverty, crowding and high population densities, the proportion of persons of color, racialized economic segregation, and low rates of health insurance exhibit increased rates of COVID‐19 cases and related deaths (Adhikari et al., 2020; Chen et al., 2020; Chin et al., 2020). Women, in particular, have experienced drastically inequitable consequences resulting from the COVID‐19 pandemic, related to medical care, systemic racial and class disparities, mental health, and motherhood (see Fulcher & Dinella, 2022). Importantly, increased unemployment rates have resulted in several negative consequences for impacted individuals and their families, including the inability to pay monthly bills, housing insecurity, food insecurity, essential service loss (e.g., internet services, phone services), loss of childcare, inability to obtain quality healthcare, and the depletion of savings (Babbar et al., 2022; Versey, 2022).

Much of the early empirical research on the COVID‐19 pandemic has been focused on factors related to rates of infection and death. More recently, empirical research has been published related to the disproportionate impacts of COVID‐19 and its inequalities associated with changes in employment and income, race and ethnicity, gender, parenting, and neighborhood characteristics (Ayoub et al., 2022; Dawson et al., 2022; Fulcher & Dinella, 2022; Garland McKinney et al., 2022; Geyton & Johnson, 2022; Jiwani et al., 2022; Rehbein et al., 2022; Versey, 2022). Additionally, research has more recently been published concentrating on uniquely vulnerable populations such as women, minorities, low socioeconomic households, and the justice‐involved community (Ayoub et al., 2022; Babbar et al., 2022; Dawson et al., 2022; Fulcher & Dinella, 2022; Garland McKinney et al., 2022; Geyton & Johnson, 2022; Heiman et al., 2022; Ibekwe‐Okafor et al., 2022; Jiwani et al., 2022; Lipp & Johnson, 2022; Rehbein et al., 2022; Versey, 2022). The present study aims to expand on the current body of literature and understand how neighborhood disorder exacerbates the socioeconomic impacts of the COVID‐19 pandemic, such as employment, housing, and access to basic necessities, particularly while taking into consideration populations especially vulnerable to socioeconomic impacts of the COVID‐19 pandemic: mothers, particularly mothers with justice‐involved sons, women of color, and women of low socioeconomic status.

## LITERATURE REVIEW

### COVID‐19 inequalities among women

#### The gender gap

Owing to several factors, the unemployment rate during the COVID‐19 pandemic is higher for women than for men (Dias et al., 2020; Kochhar, 2020). First, in the early months of the pandemic, women were more likely to be laid off than men (Dias et al., 2020). The gender gap in unemployment may be partially explained by the fact that women are more likely to be employed in the three nonfarm business sectors most harshly hit by the pandemic: leisure and hospitality, education and health services, and retail trade (Krogstad & Lopez, 2020). Second, women with young children exited the labor force at the highest rates, even higher than fathers (Landivar et al., 2020). Third, parents, particularly mothers, are vulnerable to the availability of childcare and their children's schooling to maintain their working status (Heggeness, 2020). As women with children traditionally provide most of the care and childrearing in their families (Thistle et al., 2006; Wenham et al., 2020) and many schools were forced to move to online learning for extended periods of time and many childcare facilities closed during the early months of the pandemic (Viner et al., 2020), women with children had to scale back their working hours more than fathers to meet new demands of childcare and education (Collins et al., 2021; Collins et al., 2021; Zamarro & Prados, 2021; Zarra & Ceron, 2021). This is the case even in dual‐parent homes in which both parents are capable of teleworking (Collins et al., 2021; Landivar et al., 2020; Zarra & Ceron, 2021). Indeed, mothers who lost their full‐time childcare were at increased risk of leaving their jobs in the early days of the pandemic, while childcare loss did not affect fathers’ employment (Petts et al., 2021).

#### The motherhood gap

Women with children, particularly young children, have experienced unique impacts resulting from the COVID‐19 pandemic when compared to women without children. Women with children exited the labor force at a greater rate than women without children (Landivar et al., 2020) and were more likely to take leave from work in the early months of the pandemic (Heggeness, 2020; Heggeness & Suri, 2021). In a study examining the United States Current Population Survey between September 2020 and February 2021, Lim and Zabek (2021 ) found that of women who left the work force in 2020, over half reported caregiving as the reason, further indicating the necessity of childcare and schooling for mothers to maintain employment. Women who lost their full‐time childcare during the early days of the pandemic were more likely to exit the workforce than mothers who lost less than ten hours of childcare (Petts et al., 2021). Additionally, in the United Kingdom, working parents were found to be more likely than working non‐parents to report being financially insecure during the pandemic (Cheng et al., 2021), suggesting the increased financial burden that the pandemic has put on parents.

Taken together, the present body of research suggests that women without childcare responsibilities, such as women without children and/or with older children, more easily maintained their participation in the workforce during the pandemic. It could be that women with older children that do not require childcare will experience a different level of direct responsibility for their children's health, safety, and financial support. In alignment with extant research, the present study assesses whether the socioeconomic impacts of the pandemic are experienced differently for mothers with children in the home during the pandemic than from those without children in the home. However, prior research has focused almost exclusively on how labor force participation varies among women with and without children. We expand on the current literature comparing the experiences of women with and without children by examining not only changes in employment, but other areas of socioeconomic consequence as well, such as housing, and access to food, supplies, basic utilities, and other necessary services.

#### Mothers of justice‐involved individuals

Mothers of justice‐involved individuals have been largely understudied during the COVID‐19 pandemic. While there have been multiple studies on justice‐involved women during the COVID‐19 pandemic (e.g., Beech et al., 2020; Chaimowitz et al., 2021; Ramaswamy et al., 2020), there is a gap in our knowledge regarding mothers whose children are justice‐involved, a population that is typically hard to reach. Women of justice‐involved persons experience the pandemic on their own and through their children, even when their children are adults. For example, during a pandemic, prisons and jails act as amplifiers of the disease due to overcrowded facilities and subpar living conditions (Nowotny et al., 2020). Inmates are at an increased risk of contracting COVID‐19 (Chaimowitz et al., 2021) due to limited access to preventive care, an inability to comply with physical distancing regulations, underlying health disparities, and intersecting stigmatization (Okonkwo et al., 2021). As a result, mothers of incarcerated individuals may experience emotional distress over the safety of their incarcerated child(ren). Additionally, the financial impacts of the COVID‐19 pandemic, such as rises in unemployment rates among women, may limit mothers’ ability to pay their child(ren)’s bail or aid their children in meeting various court expectations. Afterall, having justice‐involved children, whether underage or adult, is an expensive and emotional experience that alters family dynamics, is financially costly, and has spillover effects into the family's social standing in the community (Cavanagh et al., 2019). The present study utilizes a unique, understudied, and hard to reach sample of women with adult justice‐involved sons, to examine the effects of neighborhood conditions on the socioeconomic consequences of the COVID‐19 pandemic.

### Neighborhood social disorder and COVID‐19

Research on COVID‐19 and neighborhoods in the United States has focused almost exclusively on characteristics such as poverty and health insurance rates, crowding and population densities, racial and ethnic composition, and their relation to case and death numbers (e.g., Adhikari et al., 2020; Chen et al., 2020; Chin et al., 2020), but has yet to consider the effects of neighborhood disorder on individuals specifically. Neighborhood social disorder typically describes the perception or observation of social aspects of a neighborhood that signal a failure of social control (Jaśkiewicz & Wiwatowska, 2018). Social disorder is typically characterized by public behaviors such as drinking, drug use, and fighting (Jaśkiewicz & Wiwatowska, 2018; Raudenbush & Sampson, 1999; Sampson & Raudenbush, 2004; Skogan, 2004, 2012). Neighborhood poverty, individual low income, the concentration of racial and ethnic minorities, and crime rates, among others, increase the perception of neighborhood social disorder (Franzini et al., 2008; Latkin et al., 2009; Sampson & Raudenbush, 2004; Wickes et al., 2013). Neighborhood social disorder tends to be concentrated in economically disadvantaged communities, regardless of whether disorder is reported by community insiders or outsiders (Skogan, 2012).

Residents of disordered neighborhoods are at increased risk for developing various health conditions. Exposure to neighborhood disorder may result in ongoing physiological stress responses within the body, such as chronic stress and decreases in cardiovascular and neuroendocrine functioning, resulting in an increase in the likelihood of health conditions (see review by Taylor et al., 1997). Research finds that adults and children living in disordered neighborhoods report poorer overall health and mental health (both directly and indirectly; Bjornstrom et al., 2013; Hill et al., 2005; O'Brien et al., 2019; Ross & Mirowsky et al., 2001; Wallace et al., 2012). Residents of disordered neighborhoods are at high risk of obesity (Burdette & Hill, 2008; Dulin‐Keita et al., 2013; Mayne et al., 2018), cardiovascular disease biomarkers (Barber et al., 2016; Roberts et al., 2021), and childhood asthma (Vo et al., 2017), among others. Additionally, individuals living in disordered neighborhoods have limited access to health care and therefore struggle maintaining their health and receiving preventative care (Kirby & Kaneda, 2005; Zuberi & Teixeria, 2017). For example, research has found associations between high neighborhood disorder and reduced adherence to HIV treatment regimens (Surratt et al., 2015), poorer glycemic control among Type 1 diabetic adolescents (Queen et al., 2017), fewer mammograms (Davey‐Rothwell et al., 2016), and fewer dental visits (Latham‐Mintus et al., 2020) among individuals 50 years of age and above.

Importantly, many of the heath challenges associated with living in disordered neighborhoods are also risk factors for severe COVID‐19 illness (e.g., chronic kidney, liver, and lung diseases, diabetes, heart conditions, HIV, mental health conditions, obesity; see Centers for Disease Control and Prevention, 2021). As individuals with such health conditions are more likely to socially distance and leave their jobs (Couch et al., 2020) to protect their health, it can be hypothesized that residents of disordered neighborhoods, in which health risks for COVID‐19 are common, also face daunting socioeconomic impacts resulting from the pandemic. Therefore, the limited research on COVID‐19 in disordered neighborhoods leaves a gap in the literature that the present study aims to fill, by testing the extent to which greater neighborhood social disorder is associated with more economic consequences (e.g., employment, housing, food access, and utility access) of the pandemic among a diverse sample of mothers with justice involved sons.

### The present study

Women, particularly women with children, have been greatly impacted by the various socioeconomic consequences of the COVID‐19 pandemic, such as unemployment and the loss of childcare. Such consequences are likely to have been amplified for mothers living in disordered neighborhoods, where socioeconomic disadvantage (Skogan, 2012) and health risks for COVID‐19 are common (see Centers for Disease Control and Prevention, 2021). As a result, these mothers may have unique socioeconomic experiences associated with the COVID‐19 pandemic. Furthermore, socioeconomic pandemic‐related experiences of mothers differ for those with and without children in the home (Collins et al., 2021; Heggeness, 2020; Landivar et al., 2020; Lim & Zabek, 2021). Therefore, the current study asks the following research questions: (1) *Is perceived neighborhood social disorder related to socioeconomic impacts of COVID‐19 among women with justice‐involved sons?* and (2) *Does the relation between social disorder and socioeconomic impacts of COVID‐19 vary for mothers with and without children in the home?* We hypothesize that mothers who reside in neighborhoods characterized by greater social disorder will report more direct impacts of COVID‐19 than those who live in less disadvantaged neighborhoods, particularly for women with children in the home.

## METHODS

### Data

The present study utilizes data from the Crossroads Mothers Study. The Crossroads Mothers Study is a longitudinal study of mothers and female guardians of male youth who were arrested for the first time between 2011 and 2013, and who were participating in the Crossroads Study. Participants were English‐ and Spanish‐speaking primary female guardians of Crossroads youth participants in three sites: Orange County, California, Jefferson Parish, Louisiana, and Philadelphia, Pennsylvania. Mothers and/or female guardians were contacted within 1 month of their sons’ Crossroads interview for separate consent to participate in the ancillary Crossroads Mothers Study. A total of 397 women were enrolled to participate in Wave 1 of the Crossroads Mothers Study. Of eligible women, 91% consented to participate. To date, the Crossroads Mothers Study has comprised three scheduled waves of data collection (scheduled every 18 months). In March 2020, all Crossroads Mothers participants were recruited for an off‐schedule telephone interview specific to their experiences with the COVID‐19 pandemic. The pandemic‐specific interviews were completed between March 30, 2020 and August 26, 2020. A total of 221 women consented to participate in the COVID‐19 specific ancillary interview, comprising the present sample.

### Procedures

Study participants completed a 20‐min‐long telephone interview administered using computer‐assisted software with responses recorded in a secured online database. The interviews were conducted in both Spanish and English by a native speaker of the participant's chosen language. The survey materials were translated into Spanish using an iterative process through which a team of native Spanish speakers translated and back‐translated all materials to ensure the protocol was standard in both languages. This method of translation increases both cultural sensitivity and conceptual equivalence (Douglas & Craig, 2007; Khosravani & Dastjerdi, 2013). Most study participants (67.40%) completed the COVID‐19 surveys in English.

At the time of each interview, participants were informed by a trained interviewer of the purpose of the study, that participation was voluntary, and that there was no penalty for declining to participate in the pandemic‐specific survey. Participants were informed that they would be able to continue to be a part of the Crossroads Mothers Study even if they declined to participate in the COVID‐19 interview. The interviewers provided participants with a detailed explanation of the Privacy Certificate issues by the Department of Justice protecting participants’ privacy by exempting their identity and responses from subpoenas, court orders, and other disclosures. Participants were reminded of the Privacy Certificate before sensitive questions were asked. All study procedures were approved by the Institutional Review Board at the University of California, Irvine.

### Measures

#### Independent variable

**Perceived neighborhood social disorder**. In their interviews for the Crossroads Mothers Study, participants were asked to report on a Likert‐style scale from 1 (never) to 4 (always) how often they observe nine characteristics of social disorder (e.g., adults loitering/hanging out, people drinking alcohol, teens hanging out, people selling drugs, people intoxicated) in the neighborhood where they currently live (adapted from Raudenbush & Sampson, 1999). Mean scores were calculated from the participant's most recent Wave interview for the Crossroads Mothers Study to represent perceived neighborhood social disorder. Scores ranged from 1 to 4, in which higher scores correspond to higher levels of perceived social disorder. The measure displayed good reliability (*α* = .836).

#### Covariates

**Child in the home**. Mothers with children in their homes during the pandemic likely experienced the pandemic differently than those who did not, for reasons such as financial strain and time management (Collins et al., 2021; Heggeness & Suri, 2021). During their COVID‐19 surveys, participants were asked whether they currently have any childcare responsibilities, and thus at least one child under the age of 18 in their home. The measure was used as a dummy variable (0 = No Child in Home, 1 = Child in Home) in analyses conducted with the full sample and to subset the participants into two groups of mothers: those without a child in the home and those with.

**Demographics**. Participants self‐reported their age and race and ethnicity (White, Black, Latina, Other) during their baseline Crossroads Mothers Study interview. As only 3.6% (*N* = 8) of the sample reported “Other” and “Other” women were not asked to identify their race/ethnicity more specifically, the present study excluded the “Other” participants from analysis. This resulted in three racial/ethnic dummy codes: Latina, White, and Black. Latinas were used as the reference group to compare their experiences to participants of other races/ethnicities. The present study also controlled for approximate household income per month and education level as reported during the participants’ most recent Wave interview for the Crossroads Mothers Study. Education was dummy coded into two categories: less than a high school diploma, used as the reference group, and high school diploma and higher.

#### Dependent variables

**Self‐reported socioeconomic COVID‐19 impacts**. Participants were asked to report to what extent, on a Likert‐style scale from 1 (not at all impacted) to 10 (extremely impacted), they have been impacted by the COVID‐19 pandemic in each of the following domains: their job, their housing, having enough food, having enough non‐food supplies, having access to basic utilities or the internet, and having access to needed services (e.g., counseling, NA/AA, food stamps, unemployment benefits). A count variable was computed to indicate how many of the six items participants reported being impacted on, ranging from 0 to 6. The measure displayed good reliability (*α* = .856). Six binary measures were also assessed to determine whether mothers were impacted at all in each of the domains (0 = Not Impacted, 1 = Impacted) to examine the presence or absence of each impact individually. Participants with a child in the home were also asked to report the extent to which childcare or their children's schooling had been impacted during the COVID‐19 pandemic. A binary measure was computed to determine whether childcare or children's schooling was impacted at all (0 = Not Impacted, 1 = Impacted).

### Analytic plan

To address the first research question, if perceived neighborhood social disorder relates to direct socioeconomic impacts of COVID‐19, a negative binomial regression was conducted assessing the relationship between social disorder and total number of socioeconomic COVID‐19 impacts experienced. A negative binomial regression was more appropriate for analysis than a Poisson regression due to the positive skew and overdispersion of the data. Second, logistic regression analyses were conducted assessing the effect of social disorder on the individual socioeconomic impacts of COVID‐19, to determine whether social disorder was related to whether mothers were impacted in each of the area of socioeconomic impacts. The logistic regression analyses did not include the variable measuring childcare or children's schooling impact, as the question was only applicable to a portion of the study participants. All models controlled for reported importance of following CDC guidelines, age, race/ethnicity, and household monthly income. Cases with missing values were excluded from analysis (*N* = 6).

To address the second research question, if the relation between social disorder and socioeconomic impacts of COVID‐19 varies for mothers with and without children in the home, the same negative binomial and logistic regression analyses were conducted with the data subset into two samples of mothers: one with children in the home and one without children in the home. The variable measuring childcare or children's schooling impact was included in the logistic regression analysis conducted with the sample of women with children in the home only. The data was subset rather than testing for a moderation effect because we do not consider the presence or absence of children in the home as two different variations on motherhood. Rather, we believe that these are two entirely distinct experiences that are not comparable, but instead are to be considered separately.

## RESULTS

First, descriptive analyses were conducted, as presented in Table 1. In the full sample, over one half of participants were Latina (52.1%). Mean perceived neighborhood social disorder was reportedly low (1.47, *SD* = .54) and total COVID‐19 impacts was 2.89 (*SD* = 2.16). Descriptive statistics were similar in the samples with and without children currently in the home. Independent samples t‐tests indicated no significant difference in mean neighborhood social disorder [t(209) = ‐.467, *p* = .641] or mean COVID‐19 impacts [t(209) = −1.88, *p* = .061] between women with and without a child currently in the home. Significant differences appeared when examining the sample descriptive statistics by race and ethnicity. Analysis of variance tests indicated that Black participants reported greater neighborhood social disorder than Latinas and Whites, but there was no statistically significant difference between Latinas and Whites [F(2, 210) = 5.78, *p =* .004]. Additionally, analysis of variance tests indicated that Latina women report experiencing a greater number of COVID‐19 impacts than Whites [F(2, 210) = 7.27, *p =* .002]. Table 1 provides all relevant descriptive information for study participants. Second, Pearson correlations between all variables of interest are presented. Perceived neighborhood social disorder was significantly correlated with total COVID‐19 impacts, such that social disorder is correlated with greater total impacts, and greater housing, food, supplies, and utilities. Bivariate correlations are presented in Table 2.

**TABLE 1:** Sample descriptive statistics

	Descriptive statistics by child in the home status								
	Full sample (*N* = 213)			No child in the home (*N* = 99)			Child in the home (*N* = 114)		
Variable	*N* (%)	Mean (SD)	Range	*N* (%)	Mean (SD)	Range	*N* (%)	Mean (SD)	Range
Social disorder		1.47 (.54)	1‐4		1.45 (.49)	1‐3.56		1.48 (.58)	1‐4
Race/Ethnicity									
White	54 (25.4%)			29 (29.3%)			25 (21.9%)		
Black	48 (22.5%)			26 (26.3%)			22 (19.3%)		
Latina	111 (52.1%)			44 (44.4%)			67 (58.8%)		
Age		51.88 (7.19)	36‐76		53.48 (6.79)	36‐69		50.48 (7.26)	38‐76
Monthly income		$2,500 ($1,200)	$825‐≥ $12,000		$2,500 ($1,200)	$825‐≥ $12,000		$2,500 ($1,200)	$825‐≥ $12,000
Diploma	139 (64.4%)			63 (65.6%)			69 (61.6%)		
COVID impacts		2.89 (2.16)	0‐6		2.60 (2.11)	0‐6		3.15 (2.18)	0‐6
Impacted job	126 (59.2%)			59 (59.6%)			67 (58.8%)		
Impacted housing	82 (38.5%)			36 (36.4%)			46 (40.4%)		
Impacted food	103 (47.9%)			39 (39.4%)			63 (55.3%)		
Impacted supplies	122 (57.3%)			50 (50.5%)			72 (63.2%)		
Impacted utilities	86 (40.4%)			36 (36.4%)			50 (43.9%)		
Impacted services	98 (46.0%)			37 (37.4%)			61 (53.5%)		
Impacted childcare							56 (49.1%)		

	Descriptive statistics by race/ethnicity								
	Latinas (*N* = 111)			Whites (*N* = 54)			Blacks (*N* = 48)		
Variable	*N* (%)	Mean (SD)	Range	*N* (%)	Mean (SD)	Range	*N* (%)	Mean (SD)	Range
Social disorder		1.45 (.57)	1‐4		1.33 (.33)	1‐2.11		1.68 (.60)	1‐3.56
Child in home	67 (60.4%)			25 (46.3%)			22 (45.8%)		
Age		50.57 (6.87)	38‐69		54.61 (5.84)	42‐68		51.83 (8.45)	36‐76
Monthly income		$2,000 ($1,000)	$825‐≥ $12,000		$4,600 ($900)	$825‐≥ $12,000		$2,500 ($1,100)	$825‐≥ $12,000
Diploma	45 (41.7%)			53 (98.1%)			34 (73.9%)		
COVID impacts		3.35 (2.16)	0‐6		2.15 (1.81)	0‐6		2.67 (2.29)	0‐6
Job	67 (60.4%)			11 (20.4%)			28 (58.3%)		
Housing	56 (50.5%)			11 (20.4%)			15 (31.3%)		
Food	69 (62.2%)			15 (27.8%)			18 (37.5%)		
Supplies	72 (64.9%)			28 (51.9%)			22 (45.8%)		
Utilities	59 (53.2%)			10 (18.5%)			17 (35.4%)		
Services	49 (44.1%)			21 (38.9%)			28 (58.3%)		
Childcare	38 (56.7%)			2 (8%)			16 (72.7%)		

**TABLE 2:** Correlations between all variables

	1	2	3	4	5	6	7	8	9	10	11	12	13	14
1 COVID impacts														
2 Impact: Job	.50^**^													
3 Impact: Housing	.74^**^	.27^**^												
4 Impact: Food	.86^**^	.30^**^	.59^**^											
5 Impact: Supplies	.82^**^	.27^**^	.51^**^	.75^**^										
6 Impact: Utilities	.80^**^	.22^**^	.53^**^	.65^**^	.61^**^									
7 Impact: Services	.65^**^	.14^*^	.35^**^	.44^**^	.44^**^	.47^**^								
8 Impact: Childcare	.33^**^	.19	.34^**^	.28^**^	.17	.26^**^	.21^*^							
9 Social disorder	.20^*^	.03	.18^**^	.18^*^	.20^*^	.15^*^	.13	.07						
10 Child in home	.13	−.01	.04	.16^*^	.13	.08	16^*^	−	.03					
11 White	−.20^**^	−.02	−.22^**^	−.24^**^	−.06	−.26^**^	−.08	−.44^**^	−.15^*^	−.08				
12 Black	−.06	−.01	−.08	−.11	−.13	−.06	.13	−.23^*^	.21^**^	−.08	−.31^**^			
13 Age	−.26^**^	−.19^**^	−.14^*^	−.24^**^	−.22^**^	−.21^**^	−.15^*^	−.30^**^	−.08	−.21^**^	.22^**^	.00		
14 Income	−.11	.21^**^	−.20^**^	−.16^*^	−.05	−.23^**^	−.05	−.14	−.10	−.08	.45^**^	−.05	.03	
15 Diploma	−.03	.15^*^	−.12	−.10	.07	−.17^**^	.04	−.13	.03	−.04	.43^**^	.12	.10	.44^**^

*Note*: Impact: Childcare is a subset of Child in Home; thus correlation cannot be computed.

^***^p < .001, ^**^ *p* < .01, ^*^ *p* < .05

### Is perceived neighborhood social disorder related to socioeconomic impacts of COVID‐19 among women with justice‐involved sons?

Negative binomial analyses are presented in Table 3. Model 1 presents results for the full sample. The results indicated that social disorder was significantly associated with total COVID‐19 impacts, such that a one unit increase in social disorder was associated with an increase of COVID‐19 impacts by a factor of 1.25 (*p =* .014), controlling for all other variables. There was no significant difference in total COVID‐19 impacts between women with and without a child in the home. Black women reported significantly less COVID‐19 impacts than Latina women by a factor of .77 (*p* = .048).

**TABLE 3:** Negative binomial regression predicting socioeconomic COVID impacts

	Model 1: Full sample (*N* = 208)		Model 2: No child in home (*N* = 96)		Model 3: Child in home (*N* = 112)	
	B (SE)	IRR [95% CI]	B (SE)	IRR [95% CI]	B (SE)	IRR [95% CI]
Social disorder	.22^*^ (.09)	1.25 [1.05, 1.49]	.29 (.16)	1.34 [.98, 1.83]	.19 (.10)	1.21 [.99, 1.47]
Child in home	.07 (.11)	1.08 [.88, 1.33]				
White	−.37^*^ (.15)	.69 [.51, .94]	−.29 (.26)	.75 [.45, 1.24]	−.40^*^ (.19)	.67 [.46, .97]
Black	−.27^*^ (.13)	.77 [.59, .99]	−.68^**^ (.23)	.51 [.33, .79]	.06 (.16)	1.06 [.78, 1.45]
Age	−.02^**^ (.01)	.98 [.96, .99]	−.03^*^ (.01)	.97 [.95, .99]	−.02^**^ (.10)	.98 [.96, .99]
Income	−.01 (.02)	.99 [.95, 1.03]	−.03 (.03)	.97 [.91, 1.04]	−.01 (.03)	.99 [.94, 1.05]
Diploma	.17 (.12)	1.18 [.93, 1.50]	.00 (.21)	1.00 [.66, 1.50]	.31^*^ (.15)	1.37 [1.03, 1.81]
	LR *χ* ^2^ = 30.85^**^		LR *χ* ^2^ = 19.11^**^		LR *χ* ^2^ = 20.89^**^	

^***^p < .001, ^**^ *p* < .01, ^*^ *p* < .05.

Logistic regression analyses for the full sample are presented in Table 4, Model 1. Neighborhood social disorder was significantly associated with housing [*Exp(B)* = 1.98, *p =* .022], food [*Exp(B) =* 2.55, *p* = .006], and supplies [*Exp(B) =* 3.30, *p* = .006] impacts but not job, utilities, or services impacts. Compared to not having a child in the home, having a child in the home was not significantly associated with any of the binary individual COVID‐19 impacts. Compared to Latinas, White women were less likely to experience housing [*Exp(B) =* .35, *p* = .024], food [*Exp(B) = *.28, *p* = .005], and utilities [*Exp(B) =* .32, *p* = .015] COVID‐19 impacts. Black women were less likely to experience housing [*Exp(B) =* .41, *p* = .028], food [*Exp(B) =* .28, *p* = .003], supplies [*Exp(B) =* .26, *p* = .002], and utilities [*Exp(B) =* .45, *p* = .049] impacts than Latinas.

**TABLE 4:** Logistic regressions predicting individual socioeconomic COVID impacts

	Model 1: Full sample (*N* = 208)		Model 2: No child in home (*N* = 96)		Model 3: Child in home (*N* = 112)	
	Impacted job					
	B (SE)	Exp(B) [95% CI]	B (SE)	Exp(B) [95% CI]	B (SE)	Exp(B) [95% CI]
Social disorder	.11 (.29)	1.11 [.63, 1.97]	1.12^*^ (.55)	3.06 [1.03, 9.06]	−.42 (.38)	.66 [.31, 1.38]
Child in home	−.15 (.31)	.86 [.47, 1.59]				
White	−.90 (.46)	.41 [.17, 1.01]	−1.38 (.79)	.25 [.05, 1.19]	−.80 (.63)	.45 [.13, 1.54]
Black	−.32 (.42)	.73 [.33, 1.65]	−1.67^*^ (.69)	.19 [.05, .73]	.95 (.68)	2.58 [.68, 9.78]
Age	−.06^*^ (.02)	.95 [.91, .99]	−.03 (.04)	.98 [.91, 1.05]	−.09^**^ (.03)	.91 [.86, .97]
Income	.19^**^ (.07)	1.20 [1.06, 1.37]	.12 (.11)	1.12 [.91, 1.38]	.26^**^ (.10)	1.30 [1.07, 1.58]
Diploma	.68 (.38)	1.96 [.93, 4.15]	1.30 (.68)	3.10 [.82, 11.67]	.63 (.52)	1.87 [.68, 5.17]

	Nagelkerke *R* ^2^ = .14^***^		Nagelkerke *R* ^2^ = .19^*^		Nagelkerke *R* ^2^ = .27^***^	
	Impacted housing					
	B (SE)	Exp(B) [95% CI]	B (SE)	Exp(B) [95% CI]	B (SE)	Exp(B) [95% CI]
Social disorder	.69^*^ (.30)	1.98 [1.10, 3.57]	.81 (.51)	2.24 [.83, 6.06]	.65 (.38)	1.92 [.91, 4.06]
Child in home	−.02 (.32)	.98 [.53, 1.82]				
White	−1.05^*^ (.46)	.35 [.14, .87]	−.49 (.74)	.62 [.14, 2.62]	−1.48^*^ (.66)	.23 [.06, .84]
Black	−.89^*^ (.41)	.41 [.18, .91]	−1.99^**^ (.72)	.14 [.03, .55]	.00 (.54)	1.00 [.35, 2.90]
Age	−.03 (.02)	.98 [.93, 1.02]	−.02 (.04)	.98 [.91, 1.06]	−.04 (.03)	.96 [.91, 1.02]
Income	−.10 (.07)	.91 [.80, 1.03]	−.19^*^ (.11)	.82 [.67, 1.02]	−.05 (.09)	.95 [.80, 1.13]
Diploma	.18 (.37)	1.20 [.58, 2.47]	−.16 (.63)	.86 [.25, 2.91]	.47 (.48)	1.60 [.62, 4.13]

	Nagelkerke *R* ^2^ = .15^***^		Nagelkerke *R* ^2^ = .25^**^		Nagelkerke *R* ^2^ = .18^*^	
	Impacted food					
	B (SE)	Exp(B) [95% CI]	B (SE)	Exp(B) [95% CI]	B (SE)	Exp(B) [95% CI]
Social disorder	.94^**^ (.34)	2.55 [1.31, 4.98]	.70 (.53)	2.02 [.72, 5.67]	1.08^*^ (.47)	2.94 [1.18, 7.32]
Child in home	.37 (.32)	1.44 [.78, 2.68]				
White	−1.27^**^ (.45)	.28 [.12, .68]	−1.84^*^ (.76)	.16 [.04, .71]	−.87 (.57)	.42 [.14, 1.29]
Black	−1.28^**^ (.42)	.28 [.12, .64]	−2.21^***^ (.70)	.11 [.03, .43]	−.60 (.57)	.55 [.18, 1.69]
Age	−.06^*^ (.02)	.94 [.90, .99]	−.10^*^ (.04)	.90 [.83, .98]	−.04^*^ (.3)	.96 [.91, 1.02]
Income	−.06 (.07)	.94 [.83, 1.07]	−.11 (.11)	.89 [.71, 1.12]	−.04 (.09)	.96 [.81, 1.13]
Diploma	.36 (.39)	1.43 [.67, 3.07]	.43 (.67)	1.53 [.41, 5.72]	.44 (.50)	1.55 [.59, 4.11]

	Nagelkerke *R* ^2^ = .24^***^		Nagelkerke *R* ^2^ = .36^***^		Nagelkerke *R* ^2^ = .16^*^	
	Impacted supplies					
	B (SE)	Exp(B) [95% CI]	B (SE)	Exp(B) [95% CI]	B (SE)	Exp(B) [95% CI]
Social disorder	1.19^***^ (.37)	3.30 [1.59, 6.85]	1.07^*^ (.54)	2.92 [2.92, 8.44]	1.34^*^ (.55)	3.82 [1.29, 11.30]
Child in home	.26 (.32)	1.30 [.70, 2.41]				
White	−.68 (.46)	.51 [.21, 1.24]	−.60 (.72)	.55 [.14, 2.23]	−.76 (.61)	.47 [.14, 1.54]
Black	−1.35^**^ (.44)	.26 [.11, .62]	−2.34^***^ (.71)	.10 [.02, .39]	−.35 (.65)	.71 [.20, 2.51]
Age	−.07^**^ (.02)	.94 [.89, .98]	−.08^*^ (.04)	.93 [.86, .99]	−.07^*^ (.03)	.93 [.88, .99]
Income	−.05 (.07)	.95 [.83, 1.08]	−.14 (.11)	.87 [.70, 1.08]	−.01 (.09)	1.00 [.83, 1.19]
Diploma	.93^*^ (.41)	2.53 [1.13, 5.68]	.85 (.70)	2.34 [.60, 9.14]	1.16^*^ (.54)	3.20 [1.10, 9.26]

	Nagelkerke *R* ^2^ = .21^***^		Nagelkerke *R* ^2^ = .25^**^		Nagelkerke *R* ^2^ = .24^**^	
	Impacted utilities					
	B (SE)	Exp(B) [95% CI]	B (SE)	Exp(B) [95% CI]	B (SE)	Exp(B) [95% CI]
Social disorder	.58 (.31)	1.79 [.99, 3.26]	.64 (.51)	1.89 [.70, 5.11]	.49 (.38)	1.64 [.78, 3.45]
Child in home	−.06 (.32)	.94 [.50, 1.76]				
White	−1.14^*^ (.47)	.32 [.13, .80]	−1.04 (.75)	.35 [.08, 1.53]	−1.20 (.63)	.30 [.09, 1.04]
Black	−.79^*^ (.40)	.45 [.21, .99]	−1.47^**^ (.64)	.23 [.07, .80]	−.07 (.56)	.94 [.31, 2.78]
Age	−.06^*^ (.02)	.94 [.90, .99]	−.06 (.04)	.94 [.87, 1.02]	−.06^*^ (.03)	.94 [.89, .99]
Income	−.12 (.07)	.89 [.78, 1.01]	−.07 (.11)	.93 [.76, 1.14]	−.17 (.09)	.84 [.71, 1.01]
Diploma	.03 (.37)	1.03 [.50, 2.14]	−.74 (.60)	.48 [.15, 1.54]	.64 (.49)	1.90 [.72, 5.00]

	Nagelkerke *R* ^2^ = .21^***^		Nagelkerke *R* ^2^ = .29^***^		Nagelkerke *R* ^2^ = .22^**^	
	Impacted services					
	B (SE)	Exp(B) [95% CI]	B (SE)	Exp(B) [95% CI]	B (SE)	Exp(B) [95% CI]
Social disorder	.37 (.29)	1.45 [.83, 2.54]	.32 (.47)	1.37 [.55, 3.46]	.42 (.38)	1.52 [.72, 3.20]
Child in home	.58 (.30)	1.79 [.99, 3.24]				
White	−.03 (.43)	.97 [.42, 2.25]	1.61^*^ (.79)	4.99 [1.05, 23.67]	−.90 (.57)	.41 [.13, 1.24]
Black	.65 (.39)	1.91 [.88, 4.14]	1.11 (.65)	3.03 [.86, 10.75]	.75 (.60)	2.12 [.65, 3.90]
Age	−.04 (.02)	.96 [.92, 1.00]	−.08^*^ (.04)	.92 [.86, .99]	−.01 (.03)	.99 [.93, 1.04]
Income	−.05 (.06)	.85 [.85, 1.08]	−.13 (.10)	.88 [.72, 1.08]	−.04 (.09)	.97 [.82, 1.14]
Diploma	−.29 (.36)	1.34 [.66, 2.72]	−.91 (.63)	.40 [.12, 1.40]	1.05^*^ (.49)	2.86 [1.09, 7.46]

	Nagelkerke *R* ^2^ = .11^*^		Nagelkerke *R* ^2^ = .15		Nagelkerke *R* ^2^ = .15^*^	
	Impacted childcare					
					B (SE)	Exp(B) [95% CI]
Social disorder					−.06 (.38)	.94 [.45, 1.96]
White					−2.58^**^ (.83)	.08 [.02, .39]
Black					1.04 (.64)	2.82 [.81, 9.80]
Age					−.09^**^ (.03)	.92 [.86, .98]
Income					−.02 (.10)	.98 [.81, 1.19]
Diploma					.09 (.51)	1.10 [.40, 2.99]
					Nagelkerke *R* ^2^ = .37^***^	

^***^p < .001, ^**^ *p* < .01, ^*^ *p* < .05.

### Does the relation between social disorder and socioeconomic impacts of COVID‐19 vary for mothers with and without children in the home?

#### Women with no children in the home

Negative binomial analyses are presented in Table 3. Model 2 presents results for the sample of women with no children in the home (hereafter, No Child in Home; *N* = 99), In the No Child in Home model, only racial/ethnic differences and age were significant. Compared to Latinas, Black women experienced less COVID‐19 impacts (IRR = .51, *p =* .003), but no significant difference between White and Latina women was detected. Logistic regression analyses for women without children in the home are presented in Table 4, Model 2. In the No Child in Home sample, neighborhood social disorder was significantly associated with only job [*Exp(B) =* 3.06, *p* = .043] and supplies [*Exp(B) =* 2.92 *p* = .048] impacts. Compared to Latina women, White women were less likely to report food [*Exp(B) =* .16, *p* = .016] COVID‐19 impacts, but were significantly more likely to report services [*Exp(B) =* 4.99, *p* = .043] impacts. Black women were also less likely to report job [*Exp(B) =* .19, *p* = .015], housing [*Exp(B) =* .14, *p* = .005], food [*Exp(B) =* .11, *p* = .001], supplies [*Exp(B) =* .10, *p* = .000], and utilities [*Exp(B) =* .23, *p* = .021] COVID‐19 impacts than Latinas.

#### Women with children in the home

Table 3, Model 3 presents negative binomial results for the sample of women with a child in the home (hereafter, Child in Home; *N =* 114). In the Child in Home model, neighborhood social disorder did not significantly predict total COVID‐19 impacts. White women reported less COVID‐19 impacts compared to Latinas (IRR = .67, *p* = .0), while there were no significant differences between Black women and Latinas. Logistic regression analyses for women with children in the home are presented in Table 4, Model 3. Neighborhood social disorder was significantly associated with food [*Exp(B) =* 2.94, *p* = .021] and supplies [*Exp(B) =* 3.82, *p* = .016] impacts. In the Child in Home sample, race/ethnicity predicted only housing and childcare impacts, such that White women were less likely than Latinas to experience housing [*Exp(B) =* .23, *p* = .026] and childcare impacts [*Exp(B) =* .08, *p* = .002].

## DISCUSSION

The present study examined the ways in which perceived neighborhood social disorder relates to socioeconomic impacts of COVID‐19 among mothers of adult justice‐involved sons of different races and ethnicities, as well as how these relationships vary for mothers with and without children in the home. As hypothesized, the findings suggest that during the early months of the pandemic, mothers who live in neighborhoods with higher social disorder reported experiencing a greater number of socioeconomic impacts resulting from the COVID‐19 pandemic. Specifically, mothers living in neighborhoods characterized by higher social disorder were more likely to report having had their housing, food, and supplies impacted by COVID‐19. These findings may suggest that women living in disordered neighborhoods bear the brunt of the COVID‐19 pandemic due to compounded vulnerabilities, such as their race and ethnicity, socioeconomic status, justice system involvement, and various health risks associated with living in disordered neighborhoods that increase risk for COVID‐19‐related health complications.

Overall, the effect of social disorder affects socioeconomic COVID‐19 impacts above and beyond the effect of having a child in the home during the pandemic. There was no significant relationship between perceived neighborhood social disorder and total COVID‐19 impacts when the sample was subset into mothers without a child in the home and those with a child in the home. While it is possible that the small sample sizes lowered the power to detect an effect, there were also no significant differences found when comparing the effects of social disorder on COVID‐19 impacts in the full sample. In the No Child in Home sample, perceived neighborhood social disorder was not related to any of the individual COVID‐19 socioeconomic impacts but jobs and supplies. In the Child in Home sample, on the other hand, mothers living in neighborhoods with greater social disorder were more likely to report having had their food and supplies impacted by the pandemic. Taken together, the results lend to the conclusion that women with children in the home did not face greater socioeconomic impacts than those without children in the home when controlling for neighborhood social disorder.

Regarding race and ethnicity, Latinas experienced COVID‐19 socioeconomic impacts to a greater extent than mothers of other races and ethnicities, controlling for neighborhood social disorder, regardless of whether they had a child in the home. Specifically, Latina mothers in the full sample experienced a greater number of COVID‐19 socioeconomic impacts during the early months of the pandemic than both Black mothers and White mothers. Specifically, Latina mothers were more likely to report having had their housing, food, and utilities impacted by the pandemic than White and Black mothers. They were also more likely to report having had their supplies impacted than Black mothers. In the Child in Home sample, Latina mothers were significantly more likely to report having had their childcare impacted than White mothers, but there was no significant difference between Black mothers and Latina mothers in childcare impacts. This findings lends support to other research that suggests childcare impacts affect minority women greater than White women (Koltai et al., 2021). Interestingly, there were no significant differences in COVID‐19 impacts between Latinas and Black mothers in the Child in Home sample, suggesting that minority women who do have children in the home are impacted by the pandemic to a greater extent than White women with children. Afterall, people of color are more likely to face financial struggles as a result of the COVID‐19 pandemic (Versey, 2022).

There was only one instance in which White or Black mothers experienced greater impacts than Latina mothers; White mothers were more likely to report having had their access to services (e.g., counseling, NA/AA, food stamps, unemployment benefits) impacted by the COVID‐19 pandemic. Overall, this finding is supported by research that has shown that Latinx individuals are at higher risk of contracting COVID‐19 (Cervantes et al., 2021), have experienced significant economic consequences (Noe‐Bustamante et al., 2021), and are more likely to have exited the labor force (Lim & Zabek, 2021). It could be that Latinas are a vulnerable population regardless of a global pandemic, with lower employment rates (Abrego & Gonzalez, 2010; Borjas, 2017; Preston et al., 1998) and high immigrant numbers (Budiman et al., 2020; Vargas‐Willis & Cervantes, 1987) such that, for example, Latinas’ pre‐existing socioeconomic vulnerabilities predispose them for pandemic‐related socioeconomic consequences. Afterall, low‐income minorities are less likely to receive resources during emergencies, such as pandemics, than more affluent and privileged persons (Méndez et al., 2020).

### Strengths and limitations

The present study has numerous strengths. First, the present study examined the experiences of mothers with and without a child in the home individually. While a body of research has examined how the pandemic‐related experiences of women differs based on their status as mothers, such research focused almost exclusively on employment (e.g., Zamarro & Prados, 2021; Zarra & Ceron, 2021). The present study expanded on this past research by examining mothers as a whole and distinctly by their present experiences with motherhood and by examining socioeconomic consequences of the COVID‐19 pandemic beyond employment, such as housing and access to food and necessary services. Additionally, the present study utilized a sample of hard‐to‐reach, highly vulnerable women interviewed in the early months of the pandemic, expanding on the limited body of research involving women with justice‐involved children.

Despite these strengths, the present study is not without limitations. First, both neighborhood social disorder and socioeconomic impacts of COVID‐19 were obtained through self‐reports. However, research suggests that self‐report represent personal perceptions, which most accurately represent causes of actions (Junger‐Tas & Marshall, 1999). For example, Skogan (2015) describes that self‐report measures of neighborhood disorder are relatively consistent between members of the same neighborhood and remain stable over time, suggesting the benefit of using self‐report measures. Second, the participants in the current study lived in numerous cities, counties, and states during the COVID‐19 pandemic, and therefore were subjected to varying community‐level economic impacts because on COVID‐19 business, employment, and movement restrictions. We were unable to measure these differences in closures, which may have been associated with socioeconomic COVID‐19 impacts. Lastly, key characteristics of the participants, their children, and their households were not included in the data. The present sample does not include Asian mothers, who may have also experienced exacerbated consequences of the COVID‐19 pandemic if they live in disordered neighborhoods. Additionally, beyond asking participants if they had any childcare responsibilities and thus a child under the age of 18, no additional information was gathered regarding the characteristics of the children in the home. It is unknown how many children were in the home or their ages, limiting our ability to examine the differences more deeply between women with and without a child in the home. It is possible that controlling for the number and age(s) of children in the home would have allowed us to detect effects more clearly. Finally, we had no information regarding whether there were additional individuals living in the household that assisted in providing childcare or financial support. Additional assistance in the home may act as a moderator, changing the relationship between having a child in the home and COVID‐19 socioeconomic impacts.

### Future directions

While the present study aimed to understand the socioeconomic impacts of the COVID‐19 pandemic as they relate to perceived neighborhood social disorder, future research should assess the mental health impacts of the COVID‐19 pandemic among mothers living in disordered neighborhoods. Past research has found positive relationships between neighborhood disorder and maternal psychological distress (Christie‐Mizell et al., 2003; Zhang et al., 2015), negative neighborhood characteristics and maternal depressive symptoms (Hill & Herman‐Stahl , 2002), and witnessing neighborhood violence and maternal depressive symptoms (Jocson & Garcia, 2017; Jocson & Ceballo, 2020; Wilson et al., 2017). Additionally, recent research has found that in neighborhoods where services and social circles are deprived, individuals were more likely to experience adverse mental health impacts from COVID‐19 (Miao et al., 2021) and that mothers may experience increased depression during pandemic lockdowns (Aryal & Shrestha, 2020). As such, it is important to understand the relationship between neighborhood disorder and mental health among mothers in the context of the COVID‐19 pandemic.

The sample in the present study did not include men or fathers. As much previous research has found differences between men and women based on their status as parents (e.g., Heggeness, 2020; Heggeness & Suri, 2021), future research should continue to examine the experiences of men. It is likely that men and fathers living in neighborhoods characterized as highly disordered will also experience the COVID‐19 pandemic differently than men and fathers living in less disordered neighborhoods. Finally, the present study focuses on the experiences of women whose sons were previously arrested. While mothers, especially a diverse sample of mothers, are a valuable sample whose experiences are key to understanding the deep‐rooted impacts of the pandemic, it is also possible that the experiences of mothers with adult justice‐involved sons vary from mothers without justice‐involved children. Future research could also examine the experiences of women with and without justice‐system involvement.

## IMPLICATIONS AND CONCLUSIONS

The results from the present study exemplify the heightened socioeconomic impacts of the COVID‐19 pandemic in the United States among uniquely vulnerable and historically underserved populations, such as those identified in the present study as experiencing greater socioeconomic impacts from the COVID‐19 pandemic: women living in disordered and disadvantaged neighborhoods, women with children and childcare responsibilities, Latinas and other racial/ethnic minority women, and justice‐involved populations. Particular attention should also be paid to single parent households who may face severe inequity in times of need. Government and private aid agencies should acknowledge the value of equity over equality in providing relief and aid resources to individuals during pandemics and other disasters; in other words, agencies must recognize and respond to the need of such populations to receive greater and more targeted pandemic and disaster aid than those more affluent. These same parameters should be in place for those who might not normally qualify for aid, such as immigrant communities and those who are undocumented as they may face disproportionate impacts from the pandemic or disasters.

Future researchers are encouraged to understand what community outreach efforts are present in disordered neighborhoods, how these efforts have been impacted by the pandemic, and how they have affected the community. Based on the results regarding race and ethnicity in the present study, we highly encourage this focus to lie with Latina women. We also encourage future researchers to understand how these community enrichment efforts may have aided mothers during the pandemic or alleviated some of the disparities they may have faced. The examination of such efforts will further inform best practices and responses to pandemics and other disasters. Specific attention should be paid to justice‐involved individuals. Justice‐involved individuals may experience significant impacts that exacerbate the challenges they faced pre‐pandemic, such as employability and the inability to qualify for government assistance due to having a criminal background. Future research should access how these individuals have dealt with the impacts of the pandemic as well as what aid, if any, they were able to receive. Afterall, while the present study examined a sample of mothers impacted by the early months of the COVID‐19 pandemic, the results and implications can be applied to the entirety of the COVID‐19 pandemic, and to any and all other pandemics and disasters.
